# Chief medical officers’ perceptions of athlete cardiac screening

**DOI:** 10.1016/j.jsampl.2026.100138

**Published:** 2026-05-06

**Authors:** Angus J. Davis, John W. Orchard, Tim Driscoll, Jessica J. Orchard

**Affiliations:** Sydney School of School of Public Health, The University of Sydney, Australia

**Keywords:** Sports cardiology, Barrier and enablers, Sports physicians, Qualitative research

## Abstract

**Background:**

Elite athletes are screened for conditions associated with sudden cardiac arrest or death. The complexities of the real-world implementation of cardiac screening are poorly understood. The aim of this study was to understand the barriers and enablers to best practice athlete cardiac screening programs in Australia and New Zealand.

**Methods:**

Semi-structed interviews were conducted with chief medical officers (CMOs) of elite sporting organisations in Australia and New Zealand and transcribed verbatim. Interview questions were focused on CMOs’ perceptions of cardiac screening within their sporting organisation. Inductive thematic analysis was used to assign themes.

**Results:**

Twelve chief medical officers were interviewed between June 2023 and May 2024. Several themes were identified as barriers and enablers to best practice athlete cardiac screening. These themes were: 1. availability of sports cardiology expertise; 2. organisational investment in cardiac screening; 3. timing of screening; 4. accessibility of athletes; 5. athletes aged under 18; and 6. athlete engagement with cardiac screening. For CMOs, key enablers to best practice cardiac screening were access to sports cardiology expertise, together with organisational investment in the form of administrative assistance and sufficient funding to run cardiac screening. A key barrier was the organisational pressure on clinicians to make a clinical decision without sufficient clinical information, especially when screening occurred very close to competition.

**Conclusions:**

To implement best practice cardiac screening, there needs to be greater access to sports cardiology expertise, improvement in funding models, and policies guiding the timing of screening before competition.

## Graphical abstract

**Figure:**
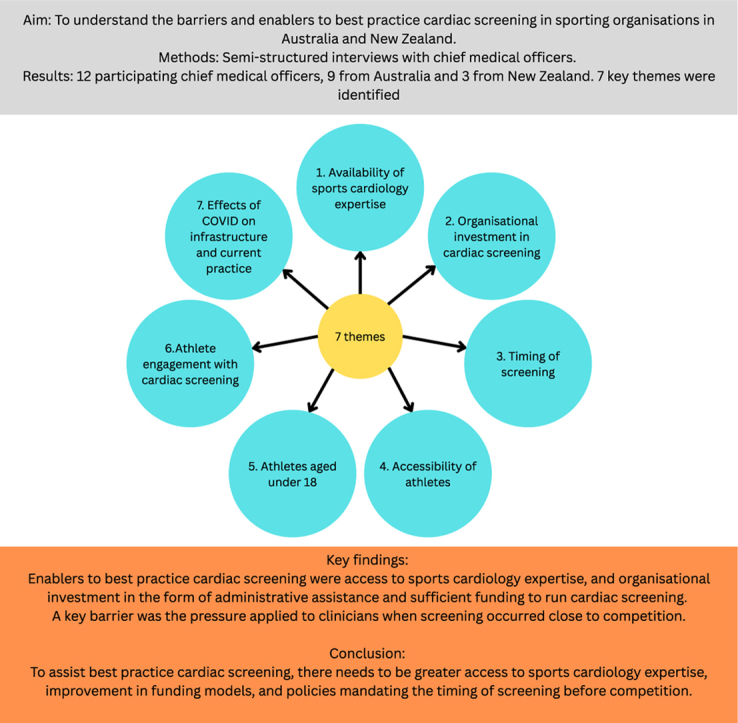

**Practical implications**

- Sports physicians require access to sports cardiology expertise for managing athlete cardiac health. This includes cardiac screening and any clinical evaluation required due to screening or the presence of symptoms.
- If organisations choose to conduct cardiac screening, they should provide administrative assistance to sports physicians to enable the effective management of the program.
- If cardiac screening is conducted by an organisation, it must be done well in order to maximise benefits and minimise any potential risks. This requires support of the organisation, sports cardiology infrastructure, ensuring ECGs are interpreted according to contemporary sport-specific criteria and that timely access to further evaluation is available for any athletes who require it.
- Cardiac screening should ideally be conducted in the off-season or at least long enough before travel to competition to allow sufficient time for appropriate follow-up if required.

## 1 Introduction

Sudden cardiac arrest or death (SCA/D) is a rare but tragic event that has a devastating effect on families and communities [1]. In athletes, SCA is the leading cause of death, at a rate of 1–2 deaths per 100,000 person years [[1], [2], [3]]. Exercise has also been identified as a trigger for SCA/D and elite athletes are exercising to the point of significant cardiac adaptation [1,[3], [4], [5], [6], [7]]. If an athlete had underlying cardiac pathology, an arrest may be precipitated by intense exercise if not appropriately managed [1,3]. Cardiac screening is recommended for elite athletes in international guidelines and by most international sporting organisations [4,[8], [9], [10], [11], [12], [13], [14], [15]]. Generally, this cardiac screening includes a personal and family history, physical examination and 12-lead resting electrocardiogram (ECG). Some sporting organisations also include an echocardiogram, for example the Federation Internationale de Football Association (FIFA) and Union Cycliste Internationale (UCI) [10,13]. Which athletes are screened depends on factors such as the level of participation and geography. In Australia and New Zealand, cardiac screening is recommended in elite athletes from a junior level and above [9]. While cardiac screening of elite athletes is well established, the real-world implementation of screening is poorly understood.

The Australasian College of Sport and Exercise Physicians (ACSEP) position statement identifies strategies for best practice cardiac screening [9]. Along with stating what should be included, the position statement emphasise the importance of governance practices and recording keeping, while also highlighting the essential involvement of sports cardiology experts. These considerations are echoed in the recent scientific statement on cardiac care of athletes from the American Heart Association and the American College of Cardiology [2].

Most sporting organisations in Australia and New Zealand have a cardiac screening policy. In a study in 2021, 19/22 chief medical officers (CMOs) of sports surveyed reported there was a formal cardiac screening policy in their sport [16]. Of those with a policy, all included history and physical examination and 89% included a 12-lead ECG. Of those that had a formal cardiac screening policy, 58% of sporting organisations paid for the cardiac screening while the rest required the athlete to pay. Given this variation, the aim of this study was to understand the barriers and enablers to implementation of best practice athlete cardiac screening programs in sporting organisations in Australia and New Zealand.

## 2 Methods

This study utilised a descriptive qualitative approach. Ethics approval was obtained from the University of Sydney Human Research Ethics Committee (Project No. 2018/649) and the report was guided by the Consolidated Criteria for Reporting Qualitative Research (COREQ) guidelines (Appendix A).

A purposive sample of CMOs of a range of elite sports across Australia and New Zealand was identified. CMOs were contacted via email and invited to participate. If they had not responded within one month, they were contacted again. Recruitment ceased when thematic saturation was reached. All participants provided informed consent before participating in the study.

A semi-structured interview prompt sheet was developed based on the study purpose and used to guide the interview process (Appendix B). Interviews began with questions designed to understand the context of cardiac screening within the CMO’s organisation, followed by questions about their perception of the screening program. Semi-structed interviews were conducted via videoconferencing (Zoom) and then transcribed verbatim by the interviewer (AJD). Participants had an existing professional relationship with the research team and the research team considered that there was no imbalance of power.

Data obtained from the semi-structed interviews were analysed inductively using thematic analysis [17]. Data familiarisation, initial coding and searching for themes was conducted by AJD. These themes were then regularly reviewed by AJD before presenting them to the author group. Through multiple discussions among all authors and multiple reviewing of themes, consensus was reached and all themes were defined, named and put in order of importance as determined by the author group.

### 2.1 Methodological rigour

Methods of data rigour were used to enhance the reliability, generalisability, and validity of the results [18]. The use of the Zoom recording feature and the use of ‘critical friends’ (JJO, TD) ensured validity and reliability of the interpretation of results. The ‘critical friends’ reviewed developing themes and interpretations, asked probing questions, and encouraged consideration of alternative explanations. Purposive sampling was used to cover a wide range of CMOs from a variety of sports to promote generalisability. Iterative consultation of the themes throughout data collection helped determine thematic saturation and guided timing to conclude data collection.

## 3 Results

Twelve interviews with CMOs were conducted between June 2023 and May 2024. The response rate was 80%. Nine CMOs were from Australia and three were from New Zealand. Interviews ran from 22 to 39 min. Seven themes were identified, with subthemes relating to barriers and enablers for implementation of best practice cardiac screening for athletes. These themes are summarised in Table 1.

**Table 1:** Themes with barriers to and enablers of best practice athlete cardiac screening.

Theme	Enabler	Barrier
1. Availability of sports cardiology expertise	Sports cardiologist available to provide advice for interpreting potentially abnormal ECGs and provide follow-up appointments as required.	Reduced access to sports cardiology expertise outside of major metropolitan areas.
2. Organisational investment in cardiac screening	Organisational support with administrators to organise the athletes and sufficient funding to have a complete screening of all eligible athletes.	Organisations not seeing screening as a medical priority.
3. Timing of screening	Screening conducted in the off-season or enough time before competition to allow sufficient time for follow-up if required.	Pressure on clinicians when screening occurs too close to competition.
4. Accessibility of athletes	Predictability of athletes' availability for screening.	Athletes who are not in Australia or New Zealand most of the time because they play an international sport. Athletes who are fringe players.
5. Athletes aged under 18	Availability of female ECG technicians helpful for junior female athletes.	Limited accessibility of athletes due to their age and potential school commitments. Age eligibility for screening.
6. Athlete engagement with cardiac screening	Athletes who are engaged with cardiac screening because they feel it provides reassurance and/or an opportunity to ask questions. Screening conducted by team physicians enables increased athlete engagement.	Some athletes (and their coaches) not seeing screening as a priority which affects their willingness to participate. Athletes not willing to complete recommended follow-up.
7. Effects of COVID-19 on infrastructure and current practice	Streamlined access to athletes with telehealth.	Fracturing of medical systems that have been difficult to rebuild.

### 3.1 Access to sports cardiology expertise

In Australia and New Zealand, sports cardiology is a sub-specialty of cardiology which encompasses various relevant cardiology subject areas including imaging, electrophysiology, inherited diseases, and genetics [19]. CMOs were assisted in conducting effective cardiac screening by access to sports cardiologists. One CMO stated:

“We were lucky in that we had this expert group set up screening” (P1).

This gave the CMO confidence in their program, knowing that they would be more likely to correctly identify abnormal ECGs and ensure an appropriate pathway to follow up if required:

“Being able to ask an expert over the phone pretty quickly and capacity to follow up in a pretty timely manner … In the absence of that, I think it's really difficult.” (P8)

Furthermore, another CMO noted:

“Really you need a sports cardiologist to be reviewing your screening ECGs. It's not enough just to review it yourself” (P11).

In some circumstances, a sports cardiologist conducted the screening rather than a sports physician, which gave CMOs confidence that the screening had been done correctly:

“I just rely on her judgment whether they can keep playing sport. So from my point of view I send them to her … and she'll tell me whether they're safe to exercise or not.” (P9)

While access to sports cardiology expertise is an enabler to best practice, some CMOs discussed the issues with access to this expertise outside of major metropolitan centres. This affected timeliness of appropriate follow-up testing, as one CMO noted:

“Unfortunately the nature of being outside some of the major capitals, access to medical specialties for everyone is challenging” (P7).

Some CMOs also noted difficulties of access to medical facilities for follow-up testing:

“The follow-up testing required for them an echo or a cardiac MRI or Holter and to see the sports cardiologist – all of that becomes exceedingly difficult if you've got them in a small country town.” (P1)

Access to sports cardiology services also affected screening policy, as noted by one CMO:

“I recommend that [sports cardiologist review each ECG]. I don't mandate because we have teams outside major metropolitan areas … so it's going to be hard for them to get that” (P10).

### 3.2 Organisational investment in cardiac screening

Administrative assistance in organisation of cardiac screening enabled best practice. Ensuring all athletes complete cardiac screening in a timely manner and attend their scheduled sessions can be complicated. Some CMOs highlighted the usefulness of medical administrators:

“They're in charge of the whole medical system and they have oversight … They would also monitor when people are due for their cardiac screening” (P7)

When the process of organising appointments and making sure everyone had appropriate cardiac screening was taken out of the hands of the CMO, it allowed them to focus on medicine. As one CMO noted:

“The things that would normally make it challenging – like the ability to get hold of the athletes – are mitigated by the high-performance manager” (P12).

Appropriate funding from the organisation to complete both screening and follow-up was an enabler in some sports. Some sporting organisations have less resources than others, which impacted the number of athletes they can screen and whether follow-up can be funded.

Some CMOs found that the allocation of funding for screening was dependant on the attitude of their organisation to screening. One CMO described their organisation’s attitude to screening:

“Within the organisation and most of the organisations I work with, [cardiac screening] is viewed as sort of being an annoyance or a luxury” (P2).

Some CMOs described how an organisations focus can be “minimise the cost” (P2), which led to decisions about who to screen being based on pragmatism rather than medicine. This frustrated those CMOs, who felt it created a barrier to athlete participation.

Funding for screening did not always include funding for follow-up. This cost then fell on the athletes in some sporting organisations:

“Follow-up is self-funded by the athlete. We don't cover that, which does create barriers in young athletes because they're very cautious, they don't want to pay” (P6).

When the cost falls on the athletes, this could result in delays in follow-up.

In Australia and New Zealand, government funded health insurance (e.g. Medicare) will not cover athlete cardiac screening but will generally cover (or partially cover) the cost further evaluation following and abnormal screening result. Private health insurance may also contribute to some costs, e.g. for a hospital procedure.

### 3.3 Timing of screening

Some organisations conduct cardiac screening very close to competition or travel. This puts time pressure on clinicians, especially if there is an abnormal result and insufficient time to complete required work-up and expert evaluation. Many CMOs noted this:

“A pre-departure camp for about three or five days – that's when we do all the cardiac screening and then if something shows up, it's a nightmare.” (P9)

“It is not good that an athlete is dealing with having an abnormal ECG and us making a decision on the morning whether or not they've got a cardiac problem that disqualifies them.” (P1)

“The thing that I find really frustrating about screenings is when we're ticking a box to screen somebody and they're going away in a week. I think that's completely the wrong thing to be doing. I think unless you've got the capacity to follow up … I think you're asking for a lot of trouble” (P8)

### 3.4 Accessibility of athletes

Several factors affected the accessibility and eligibility of athletes for cardiac screening. CMOs found management of athletes who compete internationally and fringe players complicated. An enabler was predictability of the availability of athletes for cardiac screening.

Many athletes from Australia and New Zealand compete and/or train internationally. The responsibility for their cardiac screening however often lies with the sporting organisation in their home country. This became complicated for CMOs when athletes were overseas for up to 11 months of the year:

“It is sometimes like herding cats, just because people are in all different parts of the world.” (P5).

This is further complicated when the CMO had medical responsibility for the athletes, but the sporting organisation did not directly employ the athletes:

“Most of these players we don't have full time [they are mostly overseas] and we don't have an employment relationship with them” (P2)

“A lot of our international athletes don't have any formal relationship with our national body. They're not getting funding from us, therefore we're not able to oblige them to do things except through our tournaments. This includes cardiac screening.” (P11)

Changing composition of athletic squads was also a challenge. Due to differences in cardiac screening requirements in different levels of sports there is a limit to the number of athletes who are screened in each organisation. During the season or in the lead up to competition, top athletes can be unavailable due to external factors, which means fringe players may become qualified to compete but have not had a cardiac screening. This problem was summarised by one CMO:

“Sometimes you get this perfect storm where you've got an athlete who isn't in that top tier, so they're not getting a routine screening, but they are a very good athlete and they have some degree of support, not medical. They don't have a routine screening and are then called up to compete” (P12)

### 3.5 Athletes aged under 18

Many CMOs noted difficulties with screening athletes who were under 18 and in the elite pathways of their sports. Athletes who are under 18 were often not available to participate in the routine cardiac screening for their sport and there were commonly difficulties organising their follow-up. This meant there were often short suitable screening windows prior to competition and organising follow-up needed to fit around school and other parts of young athletes’ lives:

“Following them up around school and everything else – I think that that's the group that I really struggle with” (P8).

A decision about who to screen based on their age was also complicated for CMOs. In Australia and New Zealand, recommendations suggest starting cardiac screening at 16 [20]. Some CMOs noted that this cut-off point created challenges. In sports with under 17 squads, some players may well be under 16. As one CMO put it:

“If you've got someone who's 15 in an under 17 squad, do they get screened or not?” (P1).

Finally, CMOs noted that female athletes who are under 18 may require additional considerations. One CMO highlighted the importance of having a female ECG tech for female athletes, particularly those under 18. The introduction of a policy of having a female ECG tech for female athletes improved compliance with screening in one organisation.

### 3.6 Athlete engagement with cardiac screening

Many CMOs stated that their athletes were happy with cardiac screening. This was primarily due to many seeing a normal screening result as reassuring. Doctors said that athletes:

“Have reassurance that [they are] normal”; (P1) and

“They feel safe and protected” (P2).

One CMO stated:

“[Athletes] really appreciate that there's a risk in the sport and so they're very happy to be screened” (P9).

In addition to a normal screening result, one CMO found ‘closing consultations’ a very important part of the acceptability of screening for the athletes. These are appointments after all screening has been completed where the athlete is able to ask questions about their screening result.

A benefit of cardiac screening that CMOs discussed was the opportunity for them to build rapport with the athlete outside of a regular medical context. They noted:

“The advantage that it does give the athlete and myself is that we get to know each other, before there is a pathological reason to get to know each other” (P5)

“I think screenings in general are the best opportunity to develop rapport and relationships with athletes” (P8).

CMOs found that rapport building improved future care of the athletes and enabled greater athlete engagement. Furthermore, screening was felt to be a great regular touch point with athletes which facilitated engagement throughout the rest of the year.

However, for some athletes and coaches, cardiac screening was not a priority. Some CMOs indicated that some athletes “Don’t understand the consequence of a negative screen” (P2) or see cardiac screening as a tick-box exercise which they do not see as worthwhile (P2, P5, P12). This resulted in these athletes being more unhappy with an abnormal screening result and could mean they refused to complete follow-up testing. This left athletes with an incomplete screening, placing more pressure on clinicians from the organisation to make a clinical decision without all available information.

One CMO also noted that coaches were frustrated by cardiac screening:

“For our coaches, they can see this is important. They just can't see that it's so important to give up such an amount of time for it” (P5).

### 3.7 Effects of COVID-19 on infrastructure and current practice

The COVID-19 pandemic influenced all medical services, including cardiac screening. The pandemic changed the view of athletes on cardiac health, making it more important in the eyes of clinicians and athletes in 2020 and 2021:

“An increased awareness in cardiac stuff, as a result of COVID. And there [were] pretty high cardiac concerns at one point” (P4).

In some international sports, established screening programs ceased operating due to the pandemic, as competitions stopped and many athletes were not returning home for screening.

“Medical structure became even more fragmented because we couldn't leave our country and players couldn't really come into the country … so we really lost a lot of the infrastructure we had.” (P2)

Some CMOs noted they have not returned to their pre-COVID-19 screening practices, while others have athletes who were not screened during the pandemic. They noted some gaps in their screening records and some documentation of athlete follow-up remained incomplete.

COVID-19 also led to the introduction of telehealth. Telehealth has some advantages in making follow-up conversations easier for some athletes but impacted on the ability to physically examine the patient.

## 4 Discussion

In this study, CMOs reported that access to sports cardiology expertise and organisational support were important enablers to best practice cardiac screening. Key barriers were insufficient funding and when CMOs were required to conduct cardiac screening too close to competition. CMOs also discussed the complexities of access to players, managing athletes aged under 18, athlete engagement with cardiac screening, and the effects of COVID-19.

### 4.1 Importance of sports cardiology infrastructure

Access to sports cardiology infrastructure is one of the most important aspects of implementing a cardiac screening program in athletes. Sports cardiology infrastructure is assistance from sports cardiology experts with the screening of athletes, managing their follow-up, and being part of the shared decision-making team with athletes who are diagnosed with a condition associated with SCA/D^4^. In several guidelines on cardiac screening and in the ACSEP position statement, access to appropriate expertise is considered essential for best practice [4,[8], [9], [10], [11], [12],^14^,^15^]. In this study, CMOs said that access to sports cardiology experts was very helpful in all their decision making with athletes. However, there were CMOs who did not have access to these services or were aware of clinicians within their sport who did not have this access. Sports cardiology is a growing subspecialty area, with the Cardiac Society of Australia and New Zealand establishing a working group in 2021. Outside of major metropolitan areas, there is limited access to sports cardiologists. Sports that are based in close proximity to sports cardiology centres are often well resourced, but athletes who are located outside of those areas may not have easy access to sports cardiology expertise. Given the limited availability of sports cardiologists, improvement in athlete ECG interpretation and cardiac screening by sports physicians can promote more efficient use of a scarce resource.

The necessary resources for cardiac testing are also limited. Cardiac magnetic resonance imaging may be hard to access in major metropolitan areas, with a waiting time of 2 weeks or more (or 9–12 months in some countries) not uncommon for patients. Limited access to cardiac testing extends the further away the patient is from metropolitan areas. A systematic review of eight studies assessed psychological distress of athletes after they had been screened and follow-up was complete [21]. In athletes who had a diagnosis after screening and follow-up, psychological distress increased. In athletes who either had a negative screening, or a positive screening but no cardiac diagnosis after follow-up, psychological distress was lower. It is likely that waiting for a follow-up test would cause psychological distress more similar to a true-positive diagnosis than a false-positive diagnosis. Reducing wait times for follow-up testing is therefore important for the delivery of best practice cardiac screening, and being unable to provide timely access outside of major metropolitan areas creates a health disparity [2].

### 4.2 Assistance from administrators

A complication with cardiac screening that CMOs discussed in this study was management of who needed to be screened and ensuring follow-up was completed. Sports physicians have many responsibilities when it comes to the health of athletes. Assistance from administrators in ensuring athletes attend screening sessions and in good record keeping of results were noted as extremely helpful for many participants. It allowed them to focus on medicine rather than administration.

### 4.3 Funding

CMOs reported that decisions about who had cardiac screening were primarily made based on the funding available. Cardiac screening can often be seen as a tick-box exercise as most (95–98%) athletes have a normal result [[22], [23], [24]]. Often, an abnormal screen does not lead to a diagnosis of a condition associated with SCA/D [[22], [23], [24]]. This then affects future resourcing by the sporting organisation and can result in CMOs being dissatisfied with the screening as it is not resourced sufficiently to identify and manage an athlete with a diagnosis. It has been highlighted in numerous studies that screening needs to be done to best practice standards otherwise it is not worthwhile [4,10,15,20]. This should be reflected by the provision of adequate funding. Adequate funding may be affected by budgeting or a medical salary cap, which could potentially limit a sporting organisation’s ability to adequately resource cardiac screening.

### 4.4 Timing of screening

For logistical reasons, organisations often conduct cardiac screening close to the time of competition. Internationally mandated cardiac screening does not always extend to the level below international tournaments including the events that might qualify a team or a person for that tournament [8,25]. Due to limited funding, organisations might not arrange screening for athletes who are not competing internationally. When athletes are not screened until the pre-departure camps, this creates pressure on the clinician. Ideally, screening should be done with sufficient time for follow-up to be undertaken before competition or travel.

### 4.5 Athletes aged under 18

Increasingly, athletes are in elite pathways earlier in their career than previously. This has led to discussion around the age to start screening, which was raised by CMOs in this study. Generally, the difficulty came when athletes entered an elite program before the age of 16. A recent review examining the most appropriate age to start screening found international policies on screening vary in the age to start screening [20]. In Australia and New Zealand, the position statement recommends starting at 16 and the recent review into age to start screening recommends that screening should occur no earlier than 12 years although risk of SCA/D increases by 16 [9,20]. The review also noted that along with age, the competitive level should be considered when deciding who to screen. Review of the ECG and other features of cardiac screening of an athlete who is under 16 may need to be adjusted to be more age appropriate. There are calls to have specific paediatric screening policies [26,27]. The recent paper from FIFA on younger athletes specially targets screening occurring between the ages of 12 and 16 [10], suggesting that as organisations like FIFA are adjusting their policies, significant steps are being made to consider elite athlete screening of those under 16.

### 4.6 Strengths and limitations

While there have been numerous studies on athlete perceptions of cardiac screening [21,28], to our knowledge, this is the first study of CMOs’ view of cardiac screening within their sports and has highlighted several issues for consideration.

The key limitations of the study were that not all CMOs of sports in Australia and New Zealand were interviewed. There may have been other issues related to athlete cardiac screening that were not captured in this research. However, the participating CMOs comprise a diverse sample of sports purposively sampled across Australia and New Zealand, and recruitment stopped only after saturation of issues and ideas was reached, giving the research team confidence that the key issues were highlighted by participants.

## 5 Conclusion

Cardiac screening of athletes is an important part of the care of athletes by sports physicians in Australia and New Zealand. Best practice cardiac screening by sports physicians is enabled by access to sports cardiology expertise and support from their organisation. Large barriers to best practice are insufficient funding, and timing of screening putting pressure on clinicians. To improve cardiac screening of athletes in Australia and New Zealand, there must be an increase in the number of sports cardiologists, more funding to enable organisations to support CMOs, and policies to guide the timing of screening before competition to allow sufficient time for further evaluation if required.

## Author contribution

AJD, JWO, and JJO designed the study. AJD obtained informed consent, conducted the interviews, and transcribed the interviews. AJD analysed the data, TD and JJO acted as ‘critical friends’ to ensure data trustworthiness and rigour. AJD, JWO, TD, and JJO were involved in writing the manuscript.

## Funding information

This research did not receive any specific funding.

AJD is supported by an 10.13039/100015539Australian Government Research Training Program (RTP) Scholarship through the 10.13039/501100001774University of Sydney.

JJO is supported by a 10.13039/501100000925National Health and Medical Research Council Investigator Grant (No. 2016730).

## Declaration of competing interest

The authors declare the following financial interests/personal relationships which may be considered as potential competing interests: Angus J Davis is an Associate Editor for JSAMS Plus and was excluded from any role in the review process. Jessica J Orchard is EIC for JSAMS Plus and was excluded from any role in the review process.

## Figures and Tables

**Figure:**
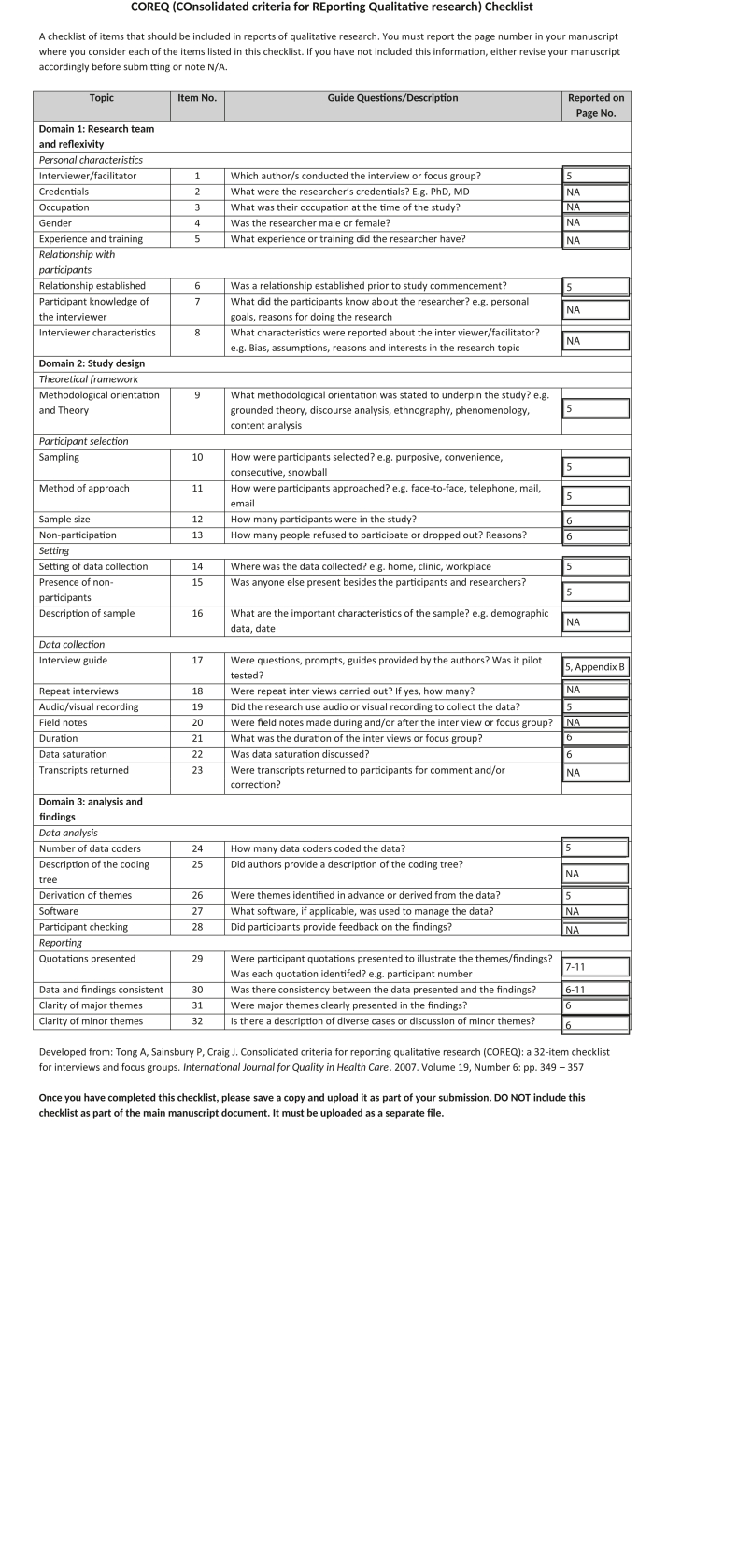


## Appendices

### Appendix B Semi-structured interview questions

1. Which levels/ages of players are screened and how often?
2. What tests are done as a standard part of the screening (ECG, echo)?
3. Logistics – are players screened all at once in the off-season, or at various times during the year?
4. Funding-who pays for the screening and follow-up tests if they are required? How much does it cost?
5. Are written waivers signed by players who decline screening?
6. How satisfied are you with the screening program overall?
7. What aspects of the screening program do you think are useful for your players?
8. What do you like about the screening program?
9. How do players respond to the screening program?
10. Does your sport have clear protocol for following up abnormal results?
11. Is there much variation between different levels of players in terms of difficulty of follow up?
12. Has your sport done any formal/informal reviews of the program and made changes? If so, what type of changes?
13. Is there anything you don’t like or find difficult with the screening program?
14. Are you likely to change your screening program in the future (e.g. introduce extra tests, screen more junior players)?
15. How did the COVID-19 pandemic effect screening?
16. Are there any other issues not covered that you would like to talk about?
